# Supplementary material for: Learning curve and surgical outcome of robotic assisted colorectal surgery with ERAS program
Source: Sci Rep. 2022 Nov 29;12:20566. doi: 10.1038/s41598-022-24665-w (PMC9709162; doi:10.1038/s41598-022-24665-w)
Supplement: Supplementary file 1 — Supplementary Information 1. [file 41598_2022_24665_MOESM1_ESM.zip › LSC Raw data-Ñ[▒K/119/O_ñw╝╨░O▒Kñσ.pdf]

正式摘要(電子簽章：000000211556571)

W52 / W52

臺中榮民總醫院

Operation Note

[redacted]

- 手術日期：2021/03/04
- 手術醫師：蔣鋒帆，CR 楊曙榮，Fellow 黃俋霖，R 翁明謙
- Anesthesia：GE
- Position：Supine
- Aseptic agents：Soap，Betadine Alcohol，Betadine Aqua

Diagnosis

- Pre-operative diagnosis : Malignant tumor of A-colon
- Post-operative diagnosis : Ditton, s/p lapascopic RH

Surgical Indication

- Removal of A-colon tumor and pathology examination

Operation

- Laparoscopic right colectomy

Finding

- 1. One 3x3cm tumor at proximal A-colon with serosa invasion
  2. Palpable enlarge LN around appendix
  3. no drain left. surgiwarp was left
  4. Moderate adhision around omentum and colon

Procedure

- Under GE anestheia, the patient was put in supine position. A 1 cm in length skin incision was made at umbilcus and the peritoneal cavity was entered and pneumoperitonium was created. The Endo-Eye Olympus laparoscope and 5mm portx3 and 12mm port was inserted. The appendix ,cecum and ileocolic vessles were identified one by one. The Ileocolic vessels was dissected, skeletonized and transectioned by clipping and Harmonic scalpel. The retroperitoneal dissection was done through embryonic plane between meso-right colon and Gerota fascia. The Duodenum was identified and protected. Lasser sac was enter and hepatic flexure was talen done. Finally, the right side colon was fully mobilized. Extracoporeal resection of ileum and T-colon with side to side anastomosis was done with GIA 75mmX2. No drain inserted and the umbilical wound was closed in layers. Throughout the whole course of operation, little blood loss and patient stood well.
- Estimated blood loss: minimum
- 器械數量: 正確
- 紗布數量: 正確

Image

- PAINT
  - abdomen

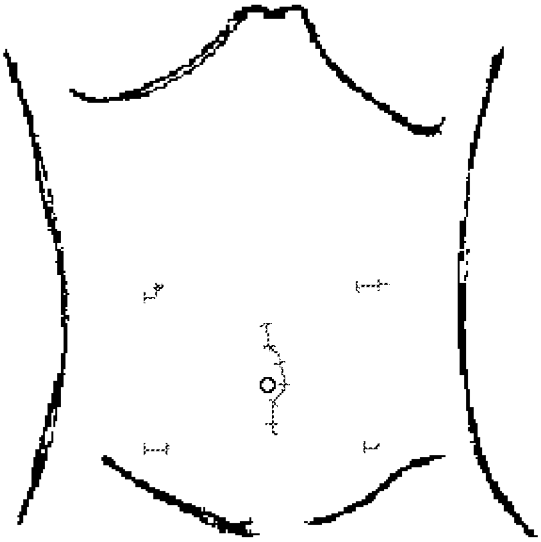

- colon & rectum

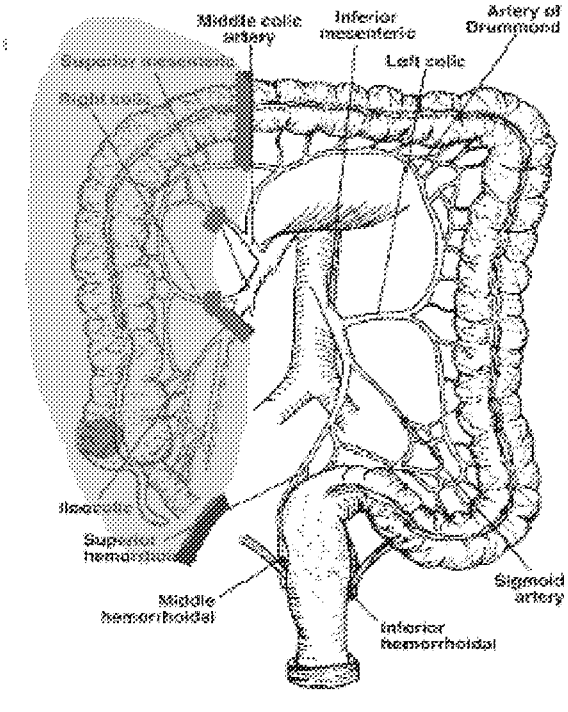

主任 2062F蔣鋒帆 / 醫師 2292G楊曙榮

正式摘要**(**電子簽章：**000000211556571)**

		W52 / W52			

doctorRank:B
